# Equity-oriented toolkit for health technology assessment and knowledge translation: application to scaling up of training and education for health workers

**DOI:** 10.1186/1478-4491-7-67

**Published:** 2009-08-05

**Authors:** Erin Ueffing, Peter Tugwell, Janet Hatcher Roberts, Peter Walker, Nadia Hamel, Vivian Welch

**Affiliations:** 1 Institute of Population Health, University of Ottawa, Ottawa, Ontario, Canada; 2 Canadian Society for International Health, Ottawa, Ontario, Canada; 3 Academy for Innovation in Medical Education, Faculty of Medicine, University of Ottawa, Ottawa, Ontario, Canada

## Abstract

Human resources for health are in crisis worldwide, especially in economically disadvantaged areas and areas with high rates of HIV/AIDS in both health workers and patients. International organizations such as the Global Health Workforce Alliance have been established to address this crisis. A technical working group within the Global Health Workforce Alliance developed recommendations for scaling up education and training of health workers. The paper will illustrate how decision-makers can use evidence and tools from an equity-oriented toolkit to scale up training and education of health workers, following five recommendations of the technical working group. The Equity-Oriented Toolkit, developed by the World Health Organization Collaborating Centre for Knowledge Translation and Health Technology Assessment in Health Equity, has four major steps: (1) burden of illness; (2) community effectiveness; (3) economic evaluation; and (4) knowledge translation/implementation. Relevant tools from each of these steps will be matched with the appropriate recommendation from the technical working group.

## Review

### The crisis in human resources for health

Human resources for health (HRH) are, arguably, the most important part of health systems [1]. HRH bring all other elements of health systems together; they link health technologies, infrastructure, knowledge, and financing [2]. Thus, when HRH are deficient, inefficient or ineffective, the entire health system is weakened; Vujicic has identified insufficient HRH capacity as one of the most significant constraints on health systems [3].

Both low-income countries (LICs) and high-income countries worldwide are experiencing a critical shortage of health workers [4], with the most dramatic crises experienced in countries with high mortality rates, reduced life expectancy and high rates of HIV/AIDS, TB, malaria and other infectious diseases [5]. A recent *World Health Report *estimates a worldwide shortage of almost 4.3 million physicians, nurses, midwives and support workers [6].

Vujicic notes that many global health initiatives are not reaching their targets because there are not enough health workers to deliver services [3]. For example, goals for immunization are not met in areas with insufficient health workers [7]. Further, a model of HRH requirements projected that Tanzania would experience a shortage of 87 100 full-time equivalent health professionals if it were to scale up priority interventions [8]. Supply is not the only problem: distribution, performance, productivity, and skill mix are also issues of concern [3].

In many African countries, HIV/AIDS not only kills health workers and reduces HRH supply, but also reduces morale and infected workers' ability to provide care, thereby reducing productivity and performance [1]. Moreover, the difficulties in working with those who have HIV/AIDS – whether colleagues or patients – may increase the willingness of health workers to move from rural areas to urban settings, from domestic/local groups to international/multilateral organizations and from care delivery to policy-making. Pull factors such as tax-free incomes, higher salaries and better working conditions have a similar impact: they draw health workers from rural to urban settings and so forth, thus exacerbating the shortages in less desirable settings.

A variety of global initiatives have been established to address the HRH crisis, including the Joint Learning Initiative [7] and the Global Health Workforce Alliance (GHWA). The Joint Learning Initiative is "a multiple stakeholder participatory process that seeks to better understand the role of workers in health systems and to identify new strategies to strengthen their performance" [7], while GHWA is a World Health Organization (WHO) group formed in 2006, with members from academia, governments, the private sector, the United Nations and other organizations. The GHWA held its first global forum for HRH in March 2008. Further, WHO has announced an initiative on task shifting [5], a process in which health care tasks are shifted to less specialized workers. This initiative was launched at the first Global Conference on Task Shifting, held in Addis Ababa in January 2008.

The call from these organizations is for a rapid scaling up of HRH capacity [3]. Further, there is a need to leverage knowledge effectively to achieve better health. Thus, within the Global Health Workforce Alliance, a Technical Working Group was tasked with developing principles and guidelines for health worker education and training scale-up; one of the authors (PW) is the Coordinator of the Technical Working Group, Task Force for Scaling Up Education and Training for Health Workers, Global Health Workforce Alliance.

In a report to WHO, the Task Force for Scaling Up Education and Training for Health Workers made recommendations for concerted action. Five of these recommendations were to:

• create a national framework for concerted action;

• create a (national) curriculum strengthening body;

• develop learning methods, materials, and approaches;

• develop the institutional action plan;

• review and evaluate process, progress and outcomes[9] [personal communication, PW].

The need to develop methods and approaches that will allow national planning authorities to address human resources inequities in the context of burden of disease and availability of effective interventions, treatment and management is crucial. Yet often the capacity to carry out such planning and the appropriateness of tools to assess such needs are lacking. Moreover, in order for an institutional action plan to be developed, decision-makers need to be assured that the plan is appropriate and needs-based. The institutional action plan also must adequately address inequities and include effective processes of evaluation to monitor progress and outcomes; outcomes should incorporate the distribution of both HRH and burden-of-illness inequities. A toolkit offering approaches and methods to address the five recommendations from the Working Group within the context of equity is the Equity-Oriented Toolkit.

### Addressing the Working Group's recommendations: the Equity-Oriented Toolkit

The World Health Organization Collaborating Centre for Knowledge Translation and Health Technology Assessment in Health Equity (available from: ; it is formerly the WHO Collaborating Centre for Health Technology Assessment) at the University of Ottawa developed a Needs-Based Toolkit for Health Technology Assessment (HTA) in collaboration with international colleagues. This toolkit was developed in response to the major recommendation of a 1993 international conference in Ottawa, "Needs-Based Technology Assessment: Exploring Global Interfaces". This meeting identified the need for the international community to develop means for developing countries to acquire the expertise to implement a needs-based approach in HTA [10].

The toolkit project was developed to assist health professionals, policy-makers and health system planners in the efficient, fair and effective allocation of health care resources, including human resources. The Technology Assessment Iterative Loop (TAIL) provided the overall framework for achieving the linkages between technology assessment and health status in a systematic manner [11]. It is needs-based according to clinical and population health needs, and therefore not "wants-based" or driven by the vested interests of health professions, industry or government. The methodology is comprehensive and consists of seven factors for assembling the information on which clinical and health policy decisions about technologies can be based. It has been developed to provide a structure to coordinate the work of a broad set of disciplines in assessing the safety, efficacy, effectiveness, costs and optimal use of technology in both populations and individual patients. The steps represent a logical progression from quantifying the burden of illness, to identifying likely causes, through to validating interventions and evaluating their efficiency, to determine whether the burden has been reduced [11].

Steps of the Needs-Based Toolkit for HTA are applicable to both the individual and to populations. The existing toolkit focused on averages, but this ignored distributional issues and equity gradients such as the impact of interventions and policies on the rich-poor gap. Averages thus ignore health inequities; that is, "differences in health which are not only unnecessary and avoidable but, in addition, are considered unfair and unjust" [12]. Averages disguise the fact that health is unevenly distributed according to socioeconomic position; health and life expectancy are significantly higher for the wealthy and decrease significantly for the poor. Furthermore, both policy and clinical interventions have been shown to be less effective for the poor and disadvantaged due to issues such as access, screening, provider compliance and consumer adherence [13].

The Needs-Based Toolkit for HTA was adapted to ensure a focus on distribution issues so that equity gradients will be detected and included in any indicators. An "equity lens" was added to focus on socioeconomic differences in health, to become what is now known as the Equity-Oriented Toolkit for HTA (EOT). The EOT is based on clinical and population health status and takes into account issues of gender equity, social justice and community participation.

The expansion into the EOT used the equity-effectiveness loop framework that assesses the consequences of reductions in efficacy in disadvantaged populations [13]. Moreover, the new EOT considered the extent to which actual tools can be used to assess the impact of health technologies on the rich-poor gap. Each tool was assessed by means of criteria that highlight the multidimensionality of the distribution of health among population subgroups. The additional innovation of this expanded toolkit is the inclusion of new advances in knowledge translation (i.e. the development and evaluation of how these tools are being used and how to make these tools transferable) to different audiences.

**Figure 1:**
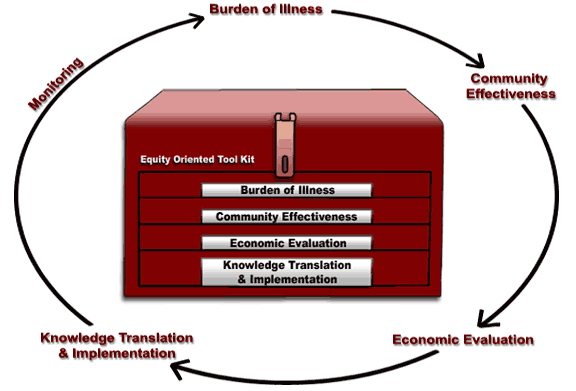
**The Equity-Oriented Toolkit**.

The EOT incorporates equity-oriented components with the following four major steps: burden of illness, community effectiveness, economic evaluation and knowledge translation and implementation (Figure 1). Each of these steps will be described, with an illustration of how the step applies to scaling up training and education.

#### Burden of illness/needs assessment

This step measures the burden of illness in a population. It incorporates both societal ("upstream") and individual ("downstream") determinants of health: cultural, genetic, political, psychosocial, environmental and biological [13]. Moreover, it also applies concepts of needs assessment and priority setting, the former helping to inform the latter. For HRH issues, the burden of illness might be measured in terms of shortages and unbalanced distributions of health workers. Thus, the results of needs assessments can be used to identify health worker coverage and prioritize plans for scaling up or redistribution of existing health workers, accordingly. Tools for needs assessment and quantifying burden of illness can also be used to assess the impact of scaling up training and education.

#### Community effectiveness

Community effectiveness describes how well an intervention will work when it is applied in the community; it may be considered the "real world" efficacy of an intervention. The interactions between five external elements determine community effectiveness: (1) efficacy; (2) screening/diagnostic accuracy; (3) health provider compliance; (4) patient adherence; and (5) coverage [13]. In the context of HRH training programmes, community effectiveness means ensuring that training programmes are efficacious, that workers needing the training are identified by means of entry requirements, that trainers and institutions comply with the agreed curricula, that students adhere to their training as required, and that training is accessible to those who need it. The toolkit provides tools that can be used to determine which educational and training interventions for health workers are effective; evidence from these tools can be used to inform scaling-up or redistribution strategies.

#### Economic evaluation

Economic evaluation describes the relationship between health benefits and costs (direct, indirect and intangible): that is, the efficiency of an intervention [13]. When applied to scaling up HRH, economic evaluation considers the cost of education and training programmes in relation to outcomes such as immunization rates or progress towards the health-oriented Millennium Development Goals. Further, economic evaluation addresses the trade-offs between equity and efficiency.

#### Knowledge translation/implementation

The Canadian Institutes of Health Research (CIHR) defines knowledge translation as "a dynamic and iterative process that includes synthesis, dissemination, exchange and ethically sound application of knowledge to improve health..., provide more effective health services and products and strengthen the health care system" [14].

### Application of the EOT

In the context of applying the EOT to HRH, the EOT insists on assessing distribution of health workers across geographical factors (e.g. rural versus urban) and sociodemographic factors (e.g. the poorest people may have less access to health workers than the least poor) associated with inequities. As these descriptions have shown, the steps of the EOT can be used to help decision-makers as they scale up HRH training and education. Tools from each step can be matched with the recommendations from the GHWA Technical Working Group [PW]; examples of appropriate tools and their applications will be described.

#### Create a national framework for concerted action via a national planning authority

According to the recommendations, a key step in scaling up training and education is to develop a national framework for concerted action, with leaders from government, international groups, public/private sectors, and civil society making shared plans[9]; we refer to this group as a national planning authority. One of the challenges – and opportunities – in establishing such a group is choosing stakeholders who will bring an appropriate blend of perspectives, experiences and opinions to the group; by including stakeholders from disadvantaged or vulnerable populations, or members of nongovernmental organizations who represent those groups as proxies, issues of equity are more likely to be addressed. An EOT tool developed by a team from Harvard can assist in this process.

That tool, PolicyMaker, "uses political mapping techniques to analyze the political actors in a policy environment. These techniques assess the power and position of key political actors, and then display the supporters, opponents and non-mobilized players in a political 'map' of the policy. This mapping provides the basis for designing strategies of political management" [15]. For scaling up, PolicyMaker can thus serve as a tool for both needs assessment (or burden of illness) and community effectiveness.

A knowledge translation/implementation tool that could also be useful for this process is the Preservice Implementation Guide from JHPIEGO, a non-profit-making health organization affiliated with Johns Hopkins University; this guide provides step-by-step directions for establishing a national working group. Moreover, the guide can also be used for each of the other Technical Working Group's recommendations and should therefore be a key resource for decision-makers addressing HRH training scale-up [16].

#### Create a national curriculum strengthening body

In addition to the planning authority, a more focused group should be formed to work on curricula and establish national standards. Walker advises that this group should include representatives from local and national training institutions in addition to external stakeholders and advisors. As when forming the planning authority, PolicyMaker can be used to determine who should be involved in this group. In Mexico, PolicyMaker was used to assess factors that influence health system reform; from this analysis, policy-makers were able to identify from which social groups – advantaged and disadvantaged – input and buy-in were crucial for success (community effectiveness) [15].

#### Develop learning methods, materials and approaches

With an appropriate planning authority and dedicated curriculum advisory group in place, specific methods and approaches should be chosen for training and education scale-up. Selecting these methods requires a reliable and strong evidence base. The Cochrane Library, maintained by the Cochrane Collaboration, is a community effectiveness tool that provides such evidence.

Formed in 1993, the Cochrane Collaboration prepares, maintains and promotes the accessibility of systematic reviews for health care [17]; it has been compared to the Human Genome Project in terms of its ambition and scale [18]. Many Cochrane reviews are applicable to both equity and the scaling up of HRH, such as reviews on recruitment strategies to increase the proportion of health workers in LMIC, rural settings and health care delivery [19,20]; specialist outreach [20]; lay health workers [21]; and integrated primary care [22]. For scaling up of education and training specifically, Cochrane reviews on audit and feedback [23], continuing medication education [24] and academic detailing (also known as educational outreach) [25] may be useful.

The Alliance for Health Policy and Systems Research (AHPSR) synthesized and summarized all systematic reviews with evidence on human resources for health for the International Dialogue on Evidence-Informed Action to achieve health goals in developing countries (IDEAHealth). They identified 26 systematic reviews, which provided evidence on training, regulatory, financial and organizational mechanisms on the supply, distribution, efficient use and performance of health workers [26]. Most of these systematic reviews (21 out of 26) assessed organizational and continuing education methods to improve the efficiency and performance of existing health workers. No evidence from systematic reviews was found to address how to design training and education curricula and programmes to increase the supply of health workers (Table 1). Lack of evidence on educational approaches may be partially due to neglecting non-health bibliographic databases such as social sciences and education.

**Table 1:** Systematic reviews on human resources for health

	**Number of systematic reviews**	**Interventions evaluated**
Training	1	Admissions criteria, curriculum content, location of training

Regulatory mechanisms	1	Recognition of overseas qualifications, underserved area service requirements

Financial mechanisms	4	Payment for performance, remuneration methods, incentives for location in underserved areas

Organizational mechanisms	21	Changes in workflow, information management, lay health workers, service integration, teamwork, substitution/extending roles, quality improvement, continuing education

Another tool useful for developing curricula is the Breakthrough Series (BTS) from the Institute for Healthcare Improvement [27]. A model for improving the quality of care, the BTS methodology addresses the gap between what we know and what we do, thus serving as a useful knowledge translation/implementation tool. Collaboratives are formed of teams from hospitals or other clinical settings who come together to address a particular issue of quality. The size of collaborative teams has ranged from 12 to 60, with each team composed of three members; these teams create a "learning system" and collaborate for six to 15 months on their quality issue [27]. Teams from schools of the health sciences could use this framework to address education and training quality at a local level, which could then be scaled up through "viral spread" without requiring substantive resources, both in terms of human capital and financing.

Financial considerations are key when establishing curricula and methods; cost-effectiveness analyses and other economic assessments provide crucial information when comparing one curriculum to another. A text by Drummond is a useful economic evaluation tool, providing methods guidelines for evaluating health care programmes [28]. These methods can also be applied to training and education strategies for HRH.

Another tool, Quermit, allows medical schools in North America (those regulated by the Liaison Committee on Medical Education) to map elements of their curricula electronically for review by the LCME. Currently, access to the information in this database is limited to the LCME; schools cannot see each other's data. However, if the access were expanded to include all medical schools, then Quermit could serve as both a burden-of-illness tool and a knowledge translation/implementation tool by allowing curriculum developers to identify gaps in their curricula and to share information with other schools on what training strategies work and what training strategies don't. Moreover, this approach could then be scaled up and adapted for other countries.

#### Develop the institutional action plan

Once curricula have been developed and training methods chosen, the planning authority and curriculum-strengthening groups must establish action plans for implementation. PolicyMaker can be used as a community effectiveness tool to determine strategic directions and inform action plans; "the software incorporates techniques of political risk analysis, in order to provide a quantitative assessment of whether a policy is politically feasible" [15]. It has been used successfully in the Dominican Republic for such a purpose, when health sector reforms were being planned by the Health Reform Group and the Inter-American Development Bank. "The analysis identified a series of political and organizational obstacles to health sector reform in that country, and assisted in the development of a strategic plan for action" [15].

PolicyMaker has also been used to develop an action plan specifically to increase the capacity of a public health system for worker training. Within an unnamed "large impoverished African country", public officials had identified that there was a significant shortage in health workers, and had recommended that more health extension workers and public health physicians be trained, particularly for rural clinics. They then used PolicyMaker to determine whether these recommendations would be accepted and would work in the community (community effectiveness) [29].

#### Review and evaluate process, progress, and outcomes

When education and training strategies are scaled up, geographical information systems (GIS; burden of illness) can be used to monitor progress through maps of HCW distribution, maps of population/HCW ratios and so forth. For example, Worldmapper is an online tool (burden of illness) that relates the size of countries to an outcome of interest, such as number of nurses working or deaths from noncommunicable diseases [30]. At the global level, Worldmapper is very useful for relative measures and provides a dramatic illustration of global disease burden; it illustrates unequal access to care and rich-poor mortality gaps. However, it does not show within-country variations or any details at lower levels. Further, the maps are only as good as the data on which they are based; Worldmapper data come from a variety of sources such as World Health Organization surveys, and thus the quality may vary depending on a country's surveillance systems and data collection. Moreover, the maps may not be updated quickly enough to effectively evaluate short-term projects.

A more responsive outcome measure may be disability-adjusted life years (DALYs), which can be used as an outcome measure to assess whether the population's burden of illness has improved with new education and training strategies. DALYs can also be used as a measure of cost-effectiveness (economic evaluation) when assessing the impact of scaling up strategies. Another economic evaluation tool, Drummond's Guidelines for Economic Submissions to the British Medicine Journal [31], can be used when developing an evaluation framework for scaling up HRH strategies; decision-makers can use this tool to inform their evaluation plans.

## Conclusion

This paper has shown that there are serious shortages and unbalanced distributions of health workers worldwide. One approach to improving the HRH situation is to address health worker training and education. The recommendations from the GHWA Technical Working Group can be used as a framework for strategies to scale up training and education. However, when policy-makers are developing these strategies, their decisions must be more than evidence-based: there is a need for evidence-informed decisions that are context-sensitive and work in real, everyday situations [32]. As illustrated in this paper, the Equity-Oriented Toolkit offers tools that can be used to assess and monitor the recommendations from GHWA, from assessing the creation of a national planning authority to evaluating the outcomes of a new education programme.

## Competing interests

EU has no known conflicts of interest. PT and JHR are the Co-Directors of the World Health Organization Collaborating Centre for Knowledge Translation and Health Technology Assessment in Health Equity; both are also members on the Coalition for Global Health Research Board. JHR serves on the Council for Foreign-Trained Graduate Nurses, and is the former Director of Migration Health for the International Organization of Migration. PW is the Coordinator of the Technical Working Group on the Task Force for Scaling Up Education and Training for Health Workers, Global Health Workforce Alliance. NH has no known conflicts of interest. VW has no known conflicts of interest.

## Authors' contributions

EU was the lead writer of the manuscript. PT developed the manuscript plan and advised on content. JHR initiated the manuscript, developed the manuscript plan and provided key figures and examples. PW provided the recommendations on which the paper is based and advised on content. NH wrote sections of the background and provided key references. VW initiated the manuscript, developed the manuscript plan and provided key examples. All authors contributed to the manuscript plan and the writing of the manuscript. All authors reviewed and approved the final manuscript.
